# The Rationale for Using Bacteriophage to Treat and Prevent Periprosthetic Joint Infections

**DOI:** 10.3389/fmicb.2020.591021

**Published:** 2020-12-21

**Authors:** Jonas D. Van Belleghem, Robert Manasherob, Ryszard Miȩdzybrodzki, Paweł Rogóż, Andrzej Górski, Gina A. Suh, Paul L. Bollyky, Derek F. Amanatullah

**Affiliations:** ^1^Division of Infectious Diseases and Geographic Medicine, Department of Medicine, Stanford University, Stanford, CA, United States; ^2^Department of Orthopaedic Surgery, Stanford University, Stanford, CA, United States; ^3^Ludwik Hirszfeld Institute of Immunology and Experimental Therapy, Polish Academy of Sciences, Wrocław, Poland; ^4^Mayo Clinic, Rochester, MN, United States

**Keywords:** periprosthetic joint infection, phage (bacteriophage), treatment, biofilm, immune system

## Abstract

Prosthetic joint infection (PJI) is a devastating complication after a joint replacement. PJI and its treatment have a high monetary cost, morbidity, and mortality. The lack of success treating PJI with conventional antibiotics alone is related to the presence of bacterial biofilm on medical implants. Consequently, surgical removal of the implant and prolonged intravenous antibiotics to eradicate the infection are necessary prior to re-implanting a new prosthetic joint. Growing clinical data shows that bacterial predators, called bacteriophages (phages), could be an alternative treatment strategy or prophylactic approach for PJI. Phages could further be exploited to degrade biofilms, making bacteria more susceptible to antibiotics and enabling potential combinatorial therapies. Emerging research suggests that phages may also directly interact with the innate immune response. Phage therapy may play an important, and currently understudied, role in the clearance of PJI, and has the potential to treat thousands of patients who would either have to undergo revision surgery to attempt to clear an infections, take antibiotics for a prolonged period to try and suppress the re-emerging infection, or potentially risk losing a limb.

## Introduction

Joint replacement is a life-enhancing procedure for millions of people around the world. Successful joint replacement improves quality of life by relieving pain as well as restoring function and independence (Giori et al., 2018). It is projected that by 2030 there will be approximately 500,000 hip and 3.5 million knee replacements performed annually in the United States alone (Kurtz et al., 2007). The vast majority of patients undergoing joint replacements experience near pain-free function, but an unfortunate minority experience pain and ultimately require additional surgery (Mortazavi et al., 2011). The etiologies of joint replacement failure include aseptic failures from loosening at the bone-cement, cement-implant, or bone-implant interfaces, fracture of the bone or implant, wear debris from the articulation, or poor implant position resulting in joint instability (Mulhall et al., 2006). However, septic failure (i.e., periprosthetic joint infection, PJI) is the most feared and often times the most common reason for joint replacement failure (Tande and Patel, 2014).

Periprosthetic joint infection is the leading cause of failure for knee replacements and the third leading cause for failure in hip replacements, accounting for between 15 and 25% of all revision surgeries (Kamath et al., 2015). Nearly 11,000 patients are affected by PJI yearly in the United States alone, costing over $1.6 billion in 2020 (Kurtz et al., 2012; Kamath et al., 2015). PJI can be categorized in three groups based on the timing of onset. Early PJI is classified when the infection occurs within 3 months after surgery, where delayed PJI occurs between 3 and 24 months after surgery. Late PJI is categorized when the PJI develops 24 months after the surgery occurred. Common signs and symptoms include swelling, redness, and pain localized to the joint, incisional erythema and/or drainage, as well as fever (Berbari et al., 1998; Bongartz et al., 2008; Ravi et al., 2012; Taylor et al., 2012).

During PJI, bacteria bound to an implant survive the administration of antibiotics by forming an antibiotic-tolerant biofilm, an extracellular polymeric substance of DNA, proteins, and polysaccharides (Fauvart et al., 2011; Urish et al., 2016). The subsequent treatment of PJI requires the removal of these biofilm contaminated implants (i.e., one- or two-stage revision surgery) in addition to the administration of antibiotics. The cost associated with each of these revisions is more than $25,000 and is associated with a significant morbidity as well as a one year mortality greater than 10% (Zmistowski et al., 2013; Kamath et al., 2015). Despite being the focus of research efforts for many years, treatment failure of PJI can be high with failure rates up between 20 and 50% when the implant is retained (Peel et al., 2011; Namba et al., 2013; Pourzal et al., 2016; Song et al., 2018).

Although PJI can occur in any patient, certain risk factors increase the risk of PJI. Obesity (body mass index, BMI > 35 kg/m^2^) was generally brought forth as a risk factor but in recent years this has been brought into question (Giori et al., 2018). Additional known factors are rheumatoid arthritis, immunosuppression, and malignancy (Berbari et al., 1998; Bongartz et al., 2008; Jämsen et al., 2009; Peel et al., 2011; Ravi et al., 2012; Taylor et al., 2012; Pourzal et al., 2016). Several studies associate PJI with poor glucose control at surgery, whereby diabetes mellitus is used as a surrogate (Malinzak et al., 2009; Cazanave et al., 2013; Namba et al., 2013; Pourzal et al., 2016). Besides disease-associated risk factors, peri-operative risk factors play an important role as well. One study has shown that hinged-knee prostheses are more frequently infected than standard replacements (Poss et al., 1984; Amanatullah et al., 2015). Additionally, postoperative complications associated with an increased risk of PJI include hematoma, superficial surgical site infection, wound drainage, and wound dehiscence (Berbari et al., 1998; Pulido et al., 2008; Aslam et al., 2010; Peel et al., 2011). Wound closure is critical, as open wounds or poorly apposed skin will more rapidly lead to bacterial colonization and subsequent infection.

Despite the existing treatment strategies for PJI such as surgical debridement and use of local and systemic antibiotics or the use of antimicrobial coatings and texturing, the presence of biofilm and the rise of antibiotic resistance limits the effectiveness of current treatment modalities. The use of bacteriophages (a.k.a., phages), viruses that specifically target bacteria, represents an alternative to therapeutic and preventative models. Understanding phage therapy begins with understanding the bacterial pathogens involved in PJI and how phage therapy can augment current treatment or prophylactic protocols.

## Bacterial Pathogenesis of PJI

Southwood et al. (1985) showed that most PJIs occurring within the first year are initiated by microorganisms introduced at the time of surgery (Popa and Dagan, 2011). This is often correlated with longer operation times (Peersman et al., 2006). Bacterial contamination occurs through either direct contact or aerosolized contamination of the prosthesis or periprosthetic tissues. Subsequently microorganisms begin colonizing the surface of the implant.

*Staphylococcus* is the predominant bacteria associated with PJIs and it likely seeds the joint as the implant crosses the skin (Table 1; Barberán, 2006; Laffer et al., 2006; Montanaro et al., 2011). Gram-positive bacteria, including *Staphylococcus aureus* and coagulase-negative *Staphylococcus* (CNS) infect between 50 and 60% of the implants (Tsukayama et al., 1996; Murdoch et al., 2001; Sendi et al., 2011). Other pathogens play an important role in PJI, including *Streptococcus* species, *Enterococcus*, and aerobic Gram-negative bacilli (Table 1; Chodos and Johnson, 2009; Lee et al., 2010; Rodríguez et al., 2010). Only a few contaminating microorganisms are needed to establish an infection and even fewer to establish a PJI. In a rabbit model, 10^4^ colony forming units (CFU) of *S. aureus* will create an infection, but when an implant is present less than 10^2^ CFU will create a PJI (Southwood et al., 1985). CNS are ubiquitous members of the human microbiome found on the skin with the most frequently identified member being *S. epidermidis* (Tripathi et al., 2020). Although less common, *Enterococcal* species account for 12–15% of early-onset PJI often as part of a polymicrobial infection (Bengtson and Knutson, 1991; Berbari et al., 1998; Cobo et al., 2011; Peel et al., 2012b; Tande and Patel, 2014).

**TABLE 1:** Overview of the most common bacterial pathogens isolated form prosthetic joint infections and their available phages for therapeutic purposes.

Infectious bacteria	Occurrence	Number of phages available (according to NCBI)	Reference of bacterial infections
*Staphylococcus aureus*	++++	145	(Berbari et al., 1998, Berbari et al., 2010; Marculescu et al., 2006; Schäfer et al., 2008; Biring et al., 2009; Lee et al., 2010; Shukla et al., 2010; Kim et al., 2011; Kusuma et al., 2011; Mahmud et al., 2012; Peel et al., 2012a)
Coagulase negative *Staphylococcus*	++++		(Berbari et al., 1998, Berbari et al., 2010; Marculescu et al., 2006; Schäfer et al., 2008; Biring et al., 2009; Lee et al., 2010; Shukla et al., 2010; Kim et al., 2011)
*Streptococcus species*	+++	55	(Berbari et al., 1998, Berbari et al., 2010; Marculescu et al., 2006; Schäfer et al., 2008; Biring et al., 2009; Lee et al., 2010; Shukla et al., 2010; Kim et al., 2011; Kusuma et al., 2011; Mahmud et al., 2012; Peel et al., 2012a)
*Enterococcus species*	++	40	(Schäfer et al., 2008; Lee et al., 2010; Shukla et al., 2010; Kim et al., 2011; Peel et al., 2012a)
*Pseudomonas aeruginosa*	+++	212	(Berbari et al., 2010; Lee et al., 2010; Shukla et al., 2010; Kusuma et al., 2011; Mahmud et al., 2012; Peel et al., 2012a)
*Escherichia coli*	++	247	(Biring et al., 2009; Lee et al., 2010; Kusuma et al., 2011; Mahmud et al., 2012)
*Acinetobacter baumannii*	+	59	(Kim et al., 2011)
*Klebsiella pneumoniae*	+	94	(Cano et al., 2020)

At times a causative bacterial pathogen cannot be isolated during PJI. The inability to grow a pathogen in laboratory culture can be attributed to prior antimicrobial treatment, inadequate use of available microbiological methods, or an inability to detect and recognized the pathogen using currently available diagnostic methods (Tande and Patel, 2014). Clearly, identifying a bacterial pathogen is critical when employing phage therapy to treat or prevent PJI. In some cases, isolation of a bacterial pathogen from the intraoperative swab may enable to prepare an active individualized phage formulation.

Another mechanism of establishing a PJI is the contiguous spread of infection from an adjacent site, called hematogenous seeding (Tande and Patel, 2014). Several studies showed that peri-operative infections at a distant site, including urinary and respiratory tract, are associated with an increased risk of PJI (Berbari et al., 1998; Peersman et al., 2001; Pulido et al., 2008). This may be the result of transient bacteremia from the distant infection site during a high-risk time period. Ultimately, however, PJI originating from remote sites of infection are rare (Popa and Dagan, 2011).

## Bacterial Biofilm

**FIGURE 1:**
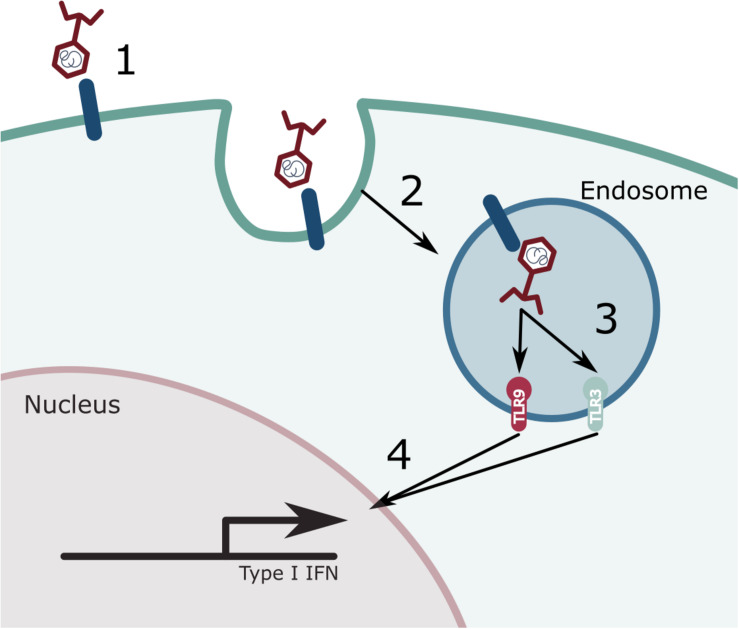
Phage induced immune responses. A variety of immune cells can recognize and interact with phages. (1) Phages are recognized by a currently unknown cell receptor. (2) This leads to the internalization of the phage where it will end up in the endosome. (3) Once present in the endosome, the phage gets degraded and its genetic content can either be recognized by TLR9 (dsDNA) or by TLR3 (dsRNA). (4) The activation of these immune receptors will lead to a signaling cascade and the expression of type I interferon (IFN).

Biofilm is part of the bacterial lifecycle in PJI (Figure 1). Biofilms are composed of an extracellular matrix made from exopolysaccharides, proteins, teichoic acids, lipids, and extracellular DNA (Arciola et al., 2012). Complex communities of bacteria are engulfed in this extracellular matrix. These communities can be mono- or polymicrobial. One of the consequences of biofilm formation during PJI is the formation of a bacterial reservoir that often leads to symptomatic but non-culturable infection, recurrent or persistent infection, or infectious spread via emboli (i.e., part of the biofilm migrates through the blood) (Azeredo and Sutherland, 2008).

The growth of a biofilm is not static but occurs through multiple stages. Starting with the attachment of the bacterial cell to a surface, followed by the initial growth on the surface, maturation of the biofilm, and finally embolization. In the end, the mature biofilm has a multicellular non-homogeneous structure wherein bacteria communicate with each other through quorum sensing. Quorum sensing make use of chemical signals to help bacteria communicate, coordinate, and cooperate. Quorum is the critical density needed to establish a biofilm colony and express virulence (Miller and Bassler, 2001; Ng and Bassler, 2009). Quorum sensing is a positive feed-forward loop which stimulates population-based gene expression (Seed et al., 1995; Rutherford and Bassler, 2012). Both Gram-negative and Gram-positive bacteria utilize these quorum sensing strategies to facilitate intraspecies communication. Additionally, due to the conserved nature of the quorum signal mechanism, inter-species communication also occurs providing a plausible explanation for cooperative polymicrobial biofilms (Mooney et al., 2018).

Bacterial sub-populations have different functions that ultimately support the whole biofilm. In this biofilm state, bacteria are protected from antimicrobials and the immune system (Donlan and Costerton, 2002). This is partially due to the physical separation of the bacteria from the antimicrobials or the immune cells, but also because bacteria are in a metabolic inactive state, called persistence (del Pozo and Patel, 2007; Molina-Manso et al., 2013). This makes the treatment of PJI with conventional antimicrobials very difficult, mandating surgical intervention, including the removal of the prothesis, to achieve a cure. Some antimicrobial agents have an effect against biofilm-resident bacteria such as rifampicin, but ultimately resistance frequently still occurs (Maudsdotter et al., 2019). Alternatively, bacteriophages, or their derived proteins can be exploited to treat biofilms.

## Bacteriophage – A Bacterial Predator

Phages, viruses that specifically target and infect bacterial cells, can be exploited to treat biofilms in PJI. Phages consist either of DNA or RNA which is encapsulated in a protein coat called a capsid (van Regenmortel, 1992). Bacteriophages are the most abundant biological entity on the world and occur everywhere in the biosphere. They have colonized even such forbidding habitats as volcanic hot springs. Their main habitats are the oceans and terrestrial topsoil (Ackermann, 2011). Phage particles can be tailed, polyhedral, filamentous, or pleiomorphic (Calendar, 2006; Ackermann, 2009). Tailed phages, representing over 96% of all known phage species, constitute the order *Caudovirales* with three families, characterized by contractile (*Myoviridae*), long and non-contractile (*Siphoviridae*), or short and non-contractile (*Podoviridae*) tails (Ackermann, 2011).

The most common phage life cycles are the lytic and lysogenic life cycle. In the lytic life cycle, the phage genome exists within the host but outside the host genome. Lytic or virulent phages repeat a cycle in which self-proliferation is synchronous with the destruction of bacteria (i.e., the lytic cycle or the virulent infection) (Matsuzaki et al., 2005). In this stage, gene expression, genome replication, and morphogenesis occurs (i.e., the formation of the genomes and capsids and the packing of the genomes in the capsids) (Ackermann, 1998). Lysogenic or temperate phages, can remain dormant in the host through integration of its genome in the bacterial genome, called a prophage, replicating along with the host until they are triggered into a lytic lifecycle. For most lysogens this trigger entails DNA damage, which can be triggered by a multitude of stimuli such as antibiotics, reactive oxygen species or UV (Ackermann, 1998; Weinbauer, 2004).

The biological characteristics of phages make them ideal for treating bacterial infections. Their lytic activity, auto-dosing, low inherent toxicity, minimal disruption of normal flora, narrow potential for inducing resistance, lack of cross-resistance with antibiotics, rapid discovery, formulation and application versatility, and biofilm clearance are characteristics looked for in antimicrobials (Loc-Carrillo and Abedon, 2011). Auto-dosing refers to the fact that phages themselves contribute to establishing the bacterial lethal dose by increasing their number during the bacterial-killing process (Carlton, 1999; Skurnik and Strauch, 2006; Chan and Abedon, 2012). A narrow host range limits the number of bacterial types with which selection for specific phage-resistance mechanisms can occur (Hyman and Abedon, 2010). Some phage derived proteins, such as endolysins or depolymerases, are able to degrade the biofilm allowing the phage to destroy the reservoir of bacteria that reside within exopolysaccharide matrix (Hanlon et al., 2001; Tait et al., 2002).

Phages are versatile in terms of formulation and can be combined with antibiotics or incorporated into scaffolds such as hydrogels or wound dressings (Alisky et al., 1998; Kutter et al., 2010; Wroe et al., 2020). They can be applied as liquids, creams, impregnated into solids, in addition to being suitable for most routes of administration (Carlton, 1999; Kutateladze and Adamia, 2010; Kutter et al., 2010). Different phages can be mixed as cocktails to broaden their properties, typically resulting in a collectively greater antibacterial spectrum of activity and lowering the chance of acquiring phage resistant bacterial strains (Merabishvili et al., 2009; Goodridge, 2010).

Phages as pharmaceuticals are protein-based, live-biological agents that can potentially interact with the body’s immune system, can actively replicate, and can even evolve during manufacture or use (Loc-Carrillo and Abedon, 2011). Phages possess unique pharmacokinetics and pharmacodynamics that remain poorly understood (Cooper et al., 2016). The pharmacokinetics of phages are complicated due to their self-replicating nature. Critical parameters that affect phage therapy are the phage adsorption rate, burst size (the number of phages released by one infected bacteria), latent period (the time between phage infection and bacterial lysis, i.e., the time needed to assemble new phage progenies), initial phage dose, and density-dependent thresholds (Payne and Jansen, 2001). Another important parameter is the clearance rate of the phage particles from the body fluids by the reticuloendothelial system. Although phages are considered generally well penetrating different tissues and body organs they may significantly differ in bioavailability after oral application (Miȩdzybrodzki et al., 2017b; Da̧browska, 2019). Their stability in environment is one of the limiting factors for production of standard phage medicinal products which require longer storage (Jault et al., 2018; Jończyk-Matysiak et al., 2019).

## Phages and the Immune System

Historically, phages were regarded as immunologically inert. However, phages do cause a humoral immune response (Ochs et al., 1971; Łusiak-Szelachowska et al., 2014; Hodyra-Stefaniak et al., 2015; Majewska et al., 2015; Zaczek et al., 2016). The production of anti-phage antibodies can affect the outcome of phage therapeutic interventions (Łusiak-Szelachowska et al., 2014). Furthermore, the route of administration plays a big role on the level of antibody production (Zelasko et al., 2016; Łusiak-Szelachowska et al., 2017). For instance, oral administration seems to lead to the lowest level of anti-phage antibodies compared to intraperitoneal injection in mouse models (Da̧browska, 2019). Moreover, low levels of anti-phage antibodies have also been detected in human subjects after oral administration of phage (Miȩdzybrodzki et al., 2017b). Although an antibody response might be present during or after a phage therapeutic intervention, this does not necessarily lead to a reduction of the therapeutic potential (Łusiak-Szelachowska et al., 2014, 2017; Zelasko et al., 2016; [Bibr B28][Bibr B29]).

However, recent research has demonstrated a phage-induced innate immune response (Figure 1). Moreover, mathematical models have predicted their importance in the outcome of a therapeutic intervention (Van Belleghem et al., 2018). As expected, this phage induced response appears to mimic an antiviral response (Van Belleghem et al., 2017; Gogokhia et al., 2019; Sweere et al., 2019). The antiviral immune response is driven by a Toll-like receptor (TLR) 9 response to *Caudovirales* DNA (Gogokhia et al., 2019) whereas it is driven by a TLR3 response to *Inoviridae* RNA (Sweere et al., 2019). The antiviral immune response may help the phage escape clearance or enable the bacterial host to thrive.

Interestingly, some phages or their preparations may exert anti-inflammatory activity (Van Belleghem et al., 2017, 2018). A significant decrease in C reactive proteins (CRP) was observed in some patients treated with phages, even in the absence of clearing the bacterial infection (Gorski et al., 2016). Moreover, it has been shown that *Escherichia coli* phage T4 presents a strong anti-inflammatory effect in mouse models reflecting the autoimmune reaction corresponding to rheumatoid arthritis (Miȩdzybrodzki et al., 2017a). These observations are in accordance with observations in humans, suggesting that phage therapy may modify the immune responses.

## Phages Aiding Suppressive Therapy

The minimal inhibitory concentration (MIC) of an antibiotic is determined on cultured bacteria and does not reflect the susceptibility of the bacteria within a biofilm. Killing the bacteria within a biofilm requires a many-fold higher concentration of antibiotic to achieve the minimum biofilm eradication concentration (MBEC) (Ricciardi et al., 2020). Thus, the use of suboptimal antibiotic concentrations could lead to antibiotic resistance in the setting of PJI. Phages are an ideal alternative or adjunct to antibiotics for treating or suppressing PJI (Table 1). Phages have a proven track record for combating, and in some cases eradicating biofilms (Tkhilaishvili et al., 2020). Even though bacteria in a biofilm, such as small colony variants, have a decreased cellular metabolic activity that often makes them resistant to antibiotics. Furthermore, studies have shown synergy between the use of systemic antibiotics and phages to treat biofilm-associated infections, although the precise mechanism is currently not known (Yilmaz et al., 2013; Kamal and Dennis, 2015; Torres-Barceló et al., 2016). On the downside, antagonistic effects between phages and antibiotics have been observed as well. *In vitro* antagonism between a mixture of two *P. aeruginosa* phages and high doses of tobramycin (Kamal and Dennis, 2015) was observed in which MBEC tobramycin was effective against *P. aeruginosa* biofilms, but its effect diminished when phage was added. This is in line with an observation that phage may sequester antibiotics, thus lowering the active concentration (Tarafder et al., 2020). Hence, when combining antibiotics and phages, strategies may have to be considered for sequential administration.

Furthermore, the occurrence of phage resistance provides an additional hurdle for the use of phage as a therapeutic (Smith and Huggins, 1983; Levin and Bull, 2004). In the therapeutic setting, these phage resistant strains can occur in 17–86% of treated patients, depending on the pathogen (Miȩdzybrodzki et al., 2012). However, phage resistance often comes at the cost of reduced bacterial virulence and can even be accompanied by re-sensitization to antibiotics (Capparelli et al., 2010; Gu et al., 2012; León and Bastías, 2015; Chan et al., 2016; Oechslin, 2018).

Only a limited amount of pre-clinical studies have evaluated the potential use of phages to treat PJI (Yilmaz et al., 2013; Kaur et al., 2016; Kishor et al., 2016; Ferry et al., 2018a, b; Cano et al., 2020), although attempts to treat PJI-like diseases, such as osteomyelitis, with phage date back to the early 1930s (Albee, 1933). Different administration routes can be deployed, as recently reviewed by Da̧browska (2019), of which oral administration or injection (intraperitoneal, intramuscular, or subcutaneous) are the most common (Da̧browska, 2019). The actual dose needed to obtain a therapeutic effect is still debated within the field with reports showing as low as 10^3^ pfu/ml being sufficient to eradicate a bacterial infection (Soothill, 1994; Marza, 2006), with general consensus saying a minimum of 10^6^ pfu/ml is needed (Morozova et al., 2018).

Phages have been used to treat PJI in the context of antibiotics (Ferry et al., 2018b). For example, in 2018, a patient with relapsing PJI of the right hip was treated by injecting a cocktail of phages into the joint in addition to systemic antibiotics. Eighteen months after phage therapy, the clinical signs of PJI were absent. This case shows the efficacy of phages in a PJI setting, although surgical intervention was still necessary for this treatment and it is unclear whether this represents suppression prior to phage resistance or eradication. Another recent case with a patient with a right total knee arthroplasty 11 years prior, suffering multiple episodes of PJI despite numerous surgeries and prolonged courses of antibiotics, showed progressive clinical worsening and development of severe allergies to antibiotics, had been offered limb amputation for his persistent right prosthetic knee infection due to *Klebsiella pneumoniae* complex. As a last resort he was offered intravenous phage therapy (Cano et al., 2020). The patient received 40 doses of a single phage spread over 8 weeks, in combination with minocycline and was able to circumvent further surgery. Furthermore, the authors were not able to identify any phage resistant strains over this eight-week course of phage treatment. This might be due to the lower metabolic activity of the bacteria in the biofilm leading to a lower chance of phage resistance to occur.

## Phages Preventing PJI

**FIGURE 2:**
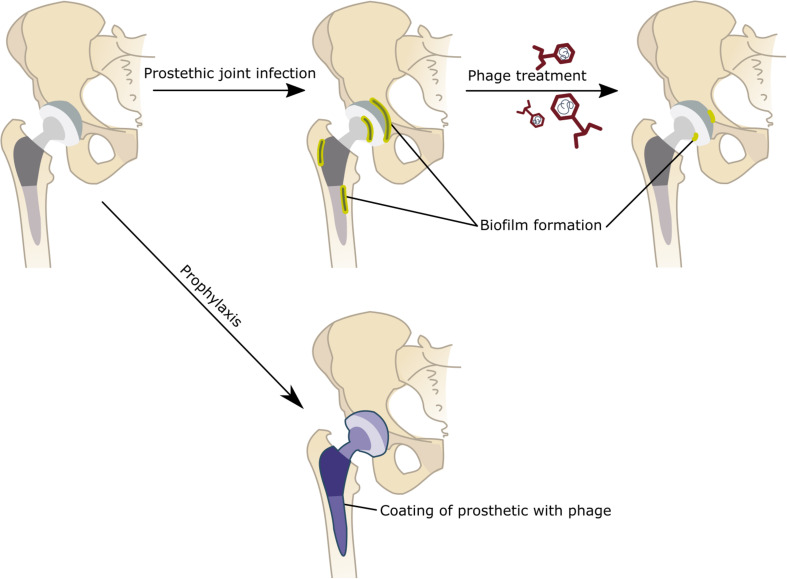
Alternative strategies for treating or preventing Prosthetic joint infections (PJI). Although that joint replacement is a life-enhancing procedure, an unfortunate minority experiences pain and will ultimately require additional surgery. Septic failure, i.e., periprosthetic joint infections, are the most common reason for joint replacement failure. During PJI, bacteria bound to the prosthetic will form biofilm structure that become resistant to antibiotic treatments. Hence the treatment of PJI requires the removal of these biofilm contaminated implants in addition to the administration of antibiotics. The use of phage can form a valid alternative (or additive to classic antibiotic treatments) to treat these PJIs without the need of a surgical intervention. These phages can be administered orally as a liquid or in a powdered formulation or injected intravenously or as a hydrogel directly in the joint. Alternatively, phages can be used to prevent the occurrence of PJI by either mixing phages in the bone cement or coating the implant with phages. In case of coating implants with phage, one could opt for a single phage or a cocktail of phages. A gradual release system could be applied, using hydroxypropyl methylcellulose (HPMC) to gradually release and deliver the therapeutic agents at the potential sites of infections. This might prevent the establishment of biofilms and the occurrence of PJIs.

Prophylactic strategies require anticipating a certain bacterial infection in order to provide the necessary agents to combat a not yet existing infection. Nevertheless, additional research is needed to further extend the lifetime of these phages after they undergo the coating or impregnation strategies to provide long lasting protection (Figure 2). It is currently not well described what amount of phage inactivation could be expected or is accepted when mixing phages with bone cement or coating them on prosthetic surfaces. Also getting a clear view of the commensal flora of a patient will become valuable in order to make educated guesses as to which phages to prevent PJI. The main difficulty with using phages in this manner is that they will lose their activity after one round of infection. This enables the removal of an initial infection but would not enable the clearance of a future recurrent infection. To tackle this problem the bioavailability of the phage can be altered by embedding them in a matrix enabling the slow release from the prosthetic bone cement over time and in different waves.

After the selective identification, patients could be decolonized of offending organisms prior to surgery, reducing surgical site infection and PJI after joint arthroplasty. The downside of the use of antibiotics for decolonization, especially in PJI, is the occurrence of antibiotic resistant strains. However, this problem would not arise when phages are used to disinfect the site of surgery. The use of phage would remove the targeted bacteria without disturbing the commensal flora or inducing dysbiosis in the patients gut or skin.

Other approaches that could be used is to directly interfere with the biofilm formation. Research has focused on disrupting biofilm formation by interfering with the quorum sensing (Sully et al., 2014; Atwood et al., 2016; Grandclément et al., 2016). These compounds target a variety of steps in the quorum sensing pathway, including the inhibition of quorum sensing signal production through degradation or substitution of SAM or acyl-ACP (precursors to acyl-homoserine lactone). Sequestration of quorum sensing signals using antibodies have also been evaluated as a potential strategy (Park et al., 2007). Alternative strategies have looked to impair the quorum signal transduction through the disruption kinase domain involved in the quorum sensing transduction (Atwood et al., 2016; Grandclément et al., 2016). Again phages could play a potential role in preventing the formation of biofilms, not due to their direct lytic activity but due to the potential effects of depolymerases present on certain phage tails (Azeredo and Sutherland, 2008; Knecht et al., 2020). These depolymerases could help degrade the biofilm matrix enabling the immune system to more effectively clear a starting bacterial infection.

The optimal form of delivery of phages to the joint implant site is unclear. Phages can be impregnated into bone cement, polymethyl methacrylate (Samokhin et al., 2018). However, once phages are impregnated into polymethyl methacrylate they lose their effective titer between over 1–2 weeks.

Recent research points into the potential of using phages to coat prosthetic materials. Different strategies could be applied, one could coat with a single phage or a phage cocktail either to one specific pathogen or a diverse set of pathogens. Kaur et al. (2016), showed the potential of using phage and antibody coated Kirshner wire to prevent *S. aureus* infection when used at the site of the prosthetic (Kaur et al., 2016). The authors used a hydroxypropyl methylcellulose (HPMC) gel, for the gradual release and delivery of two therapeutic agents at the implant site. These coatings of the Kirshner wires remained stable over 20 days, although a 3-log reduction could be observed after initial coating. Moreover, the authors observed that the elution from this gel remained steady for 48 h. A combinatorial approach of the *S. aureus* phage and linezolid led to a reduction of bacterial adhesion by 4-log, as well as reducing the occurrence of phage resistant strains when phage alone was used (Kaur et al., 2016).

## Conclusion

Orthopedic devices are prevalent and durable making them one of the most common surgical implant types to become infected (Inzana et al., 2016). All of the materials used for implantable orthopedic devices are easily colonized by bacteria (Gbejuade et al., 2015). Nevertheless, several preventative and therapeutic strategies exist, some more invasive then others. Bacteriophage is a valuable addition as the field looks to control antimicrobial infection in a more effective manner – moving beyond the morbidity of the scalpel and delivering higher doses of resistance-generating antibiotics. A very promising path is the use of phage coated prosthetics. Although the pitfall here lies in the fact that the immobilized phage will lose its activity after one round of infection. This would still allow to combat an initial infection but not a recurrent one. To tackle this problem the bioavailability of the phage can be altered by embedding them in a matrix enabling the slow release from the prosthetic bone cement over time and in different waves. Alternatively, phage embedded in hydrogels and injected directly at the site of infection could be performed on patients that already have an implant to remove the infection. This would enable to physicians to treat PJIs without the need of surgical intervention or removal of the implant, providing layover between the development of phage coated prosthetics and the use of phage in PJI. The use of phages could also enable to combat current unculturable bacteria, on the condition that they can easily be identified through genomic approach. It has been suggested that machine learning approaches can be utilized to either identify, or generate through synthetic genomics, based on the genomic information provided on the bacterial target (Leite et al., 2018; Martorell-Marugán et al., 2019; Baláž et al., 2020; Pirnay, 2020).

Nevertheless, to use phages under these circumstances the field needs to further invest in understanding the bioavailability and biodistribution of phages as well as their immunogenicity in order to generate the best outcome for the patients. Although rigorous clinical trials are currently lacking progress has begun to treat PJI with phage.

## Author Contributions

All authors participated in the conception, drafting, and/or editing of the manuscript.

## Conflict of Interest

The authors declare that the research was conducted in the absence of any commercial or financial relationships that could be construed as a potential conflict of interest.
